# Artificial Intelligence Algorithm-Based MRI for Differentiation Diagnosis of Prostate Cancer

**DOI:** 10.1155/2022/8123643

**Published:** 2022-06-28

**Authors:** Rui Luo, Qingxiang Zeng, Huashan Chen

**Affiliations:** ^1^Department of Radiology, Xiangyang Central Hospital, Affiliated Hospital of Hubei University of Arts and Sciences, Xiangyang, 441021 Hubei, China; ^2^Department of Group Work, Xiangyang Central Hospital, Affiliated Hospital of Hubei University of Arts and Sciences, Xiangyang, 441021 Hubei, China

## Abstract

The rapid increase in prostate cancer (PCa) patients is similar to that of benign prostatic hyperplasia (BPH) patients, but the treatments are quite different. In this research, magnetic resonance imaging (MRI) images under the weighted low-rank matrix restoration algorithm (RLRE) were utilized to differentiate PCa from BPH. The diagnostic effects of different sequences of MRI images were evaluated to provide a more effective examination method for the clinical differential diagnosis of PCa and BPH. 150 patients with suspected PCa were taken as the research objects. Pathological examination revealed that 137 patients had PCa and 13 patients had BPH. The pathological results were the gold standard and were compared with the MRI results of different sequences. Therefore, the accuracy of the MRI results was evaluated. The results showed that with the rise of Gaussian noise, the peak signal-to-noise ratio (PSNR) and structural similarity (SSIM) of all three algorithms gradually decreased, but the PSNR and SSIM of the RLRE algorithm were always higher than those of the RL and BM3D algorithms (*P* < 0.05). The sensitivity (97.08%), specificity (92.31%), accuracy (96.67%), and consistency (0.678) of the dynamic contrast enhancement (DCE) sequence were higher than those of the plain scan (86.13%, 69.23%, 84.67%, and 0.469, respectively). In conclusion, the RLRE algorithm could promote the resolution of MRI images and improve the display effect. DCE could better differentiate PCa from BPH, had great clinical application value, and was worthy of clinical promotion.

## 1. Introduction

As a common genital malignancy, the incidence of prostate cancer (PCa) increases with age. Moreover, with the development of society, the Chinese population is aging seriously, and the number of PCa patients is also rising sharply. As the incidence is 1.6/100,000 and the mortality is 1.0/100,000, PCa is more common in older men [1, 2]. PCa causes great damage to the physical and mental health of middle-aged and elderly men and even endangers the lives of patients. The main clinical manifestations of PCa are frequent micturition, urgency, dysuria, hematuria, and urinary retention. Benign prostatic hyperplasia (BPH) has many similarities with PCa in onset age and clinical manifestations, and the pathogenesis of some PCa patients is BPH [3, 4]. However, there is a great difference between them in treatment methods. Therefore, the differential diagnosis of PCa and BPH disease is very important in the early stage.

There are many clinical diagnostic methods for PCa and BPH, including digital rectal examination, ultrasonography, prostate-specific antigen, needle biopsy, and magnetic resonance imaging (MRI) [5]. However, digital rectal examination is not intuitive; ultrasound has a high rate of missed detection due to its imaging principle, and prostate-specific antigen examination has abnormal results [6]. Needle biopsy is the “gold standard” for malignant tumor diagnosis, but invasive operation will cause pain to patients, so it is not easily adopted [7]. MRI is a widely recognized diagnostic method for benign and malignant prostate diseases because of its simple operation, noninvasiveness, high image resolution, lack of radiation, and multiple scan sequences [8, 9]. MRI has important application value for evaluating prostate diseases. It can only detect the infringement condition and lesions from various angles and thus assists doctors in accurate radiotherapy, avoiding involving other tissues [10–12]. However, MRI images often have artifacts due to the patients' inability to remain still for the long scanning time, which affects the clarity of images.

Whether medical images can clearly show effective information about the disease is very important for the diagnosis of the disease. Therefore, the improvement of image quality has become the focus of attention. With the rapid development of artificial intelligence, a large number of intelligent algorithms have been used to improve the imaging quality of medical images [13, 14]. Studies have shown that intelligent algorithms have a significant effect on denoising MRI images [15]. Among them, the low-rank matrix restoration algorithm [16] has become a research hotspot in the field of image processing due to its stability. However, studies have found that the image processed by this algorithm has fringe distortion, and the denoising effect decreases sharply when the noise increases [17]. Then, the weighted low-rank matrix restoration algorithm (RLRE for short) was proposed, and it was found that the algorithm had a significant denoising effect and significantly alleviated the above problems [18].

In summary, in this study, MRI images were processed by the RLRE for differential diagnosis between PCa and BPH patients, and needle biopsy was used as the gold standard to evaluate the application value of different MRI sequences in differential diagnosis between PCa and BPH diseases, which is expected to provide a basis for the clinical diagnosis of malignant prostate tumors.

## 2. Materials and Methods

### 2.1. Research Subjects

In this study, 150 patients with suspected PCa were included as the research objects, as they went to the hospital for diagnosis and treatment from January 2018 to April 2021. They ranged in age from 24 to 80 years, with an average age of 51.34 ± 8.72 years old. The prostate mass ranged from 28 g to 105 g, with an average prostate mass of 63.12 ± 12.22 g. There were 93 patients with dysuria, 80 patients with urgency and pain, and 50 patients with hematuria. Pathological examination showed 137 patients with PCa and 13 patients with BPH. All the patients were examined with different sequences of MRI, and the images were denoised with the RLRE algorithm. Afterwards, the pathological examination results were taken as the gold standard and compared with the test results of different MRI sequences to evaluate the diagnostic performance of different MRI sequences for PCa. This study was approved by ethics committee of hospital, and informed consents were obtained from patients.

Inclusion criteria were as follows: (a) all patients were over 18 years old; (b) patients received pathological examinations by needle biopsy, and the results met the diagnostic criteria for PCa and BPH [19]; (c) patients whose pathological biopsy results suggested PCa or BPH; (d) patients tested by MRI with different sequences; and (e) patients who had signed informed consent.

Exclusion criteria were as follows: (a) patients with serious organ dysfunction, such as heart, liver, and kidney dysfunction; (b) patients with other systemic cancer diseases; (c) claustrophobic patients; (d) patients implanted with metal objects; (e) patients allergic to contrast agents; and (f) patients with poor image quality or incomplete clinical data.

### 2.2. MRI Examination

The patient was asked to take the supine position, and the abdominal phase-controlled coil of a 1.5T superconducting MRI scanner was fixed at the superior margin of the pubic symphysis. Plain scanning, diffusion weighted imaging (DWI), and dynamic contrast enhancement (DCE) scanning were performed on the patients, and the specific scanning parameters were as follows:

1. Parameters of plain scan sequence: T1WI sequence, axial position, and time of repetition (tr) = 500 ms, time of echo (te) = 8 ms, T2WI sequence, axial position, and TR = 3330 ms, TE = 80 ms, slice thickness = 5 mm, visual field = 21 cm × 21 cm, and matrix = 520 × 520, with scanning range from iliac artery bifurcation level to pelvic floor
2. DWI sequence, axial position, and TR = 2500 ms, TE = 60 ms, layer thickness = 2.5 mm, field of vision = 21 cm × 21 cm, matrix = 130 × 130, and *B* value was set as 0/800 s/mm^2^, with the scanning range of the whole prostate and seminal vesicle
3. DCE sequence, axial position, and TR = 9.8 ms, TE = 5.2 ms, layer thickness = 4.5 mm, field of vision = 21 cm × 21 cm, and matrix = 252 × 252. The scanning range was the whole prostate and seminal vesicle. Before scanning, 0.2 mmol/kg gadolinium meglumine and 20 mL normal saline were injected for delayed enhanced scanning [20]

The image results obtained from the above scans were imported into the MRWP postprocessing workstation, and two experienced and senior doctors (more than 25 years) reviewed the images without knowing the pathological examination results.

### 2.3. Image Denoising Algorithm Based on Weighted Low-Rank Matrix Restoration

#### 2.3.1. Algorithm Application

The RLRE introduces the constraint term of Gaussian noise based on the traditional low-rank matrix restoration algorithm (RL) and adds in the weighted low-rank matrix and sparse local matrix to solve the problems of RL. The expression of the algorithm can be expressed as follows.

(1) $$\begin{array}{c} \underset{H,S,E}{\min}\overset{n}{\underset{j=1}{\sum }}w_{H,j}•\sigma_{j}+\lambda_{1}{\left\|W_{s}•S\right\|}_{1}+\lambda_{2}{\left\|E\right\|}_{F}^{2} \end{array}$$

(2) $$\begin{array}{c} \text{s}.\text{t}.H+S+E=D \end{array}$$

where *S* and *H* represent the matrix, *W*_*H*_ = {*w*_*H*,*j*_} and *W*_*s*_ represent the singular value weight of matrix *H* and matrix *S*, *σ*_*j*_ represents the singular value of matrix *H*, and *λ*_2_‖*E*‖_*F*_^2^ represents the constraint term of Gaussian noise.

To solve the above equation, we first analyze *W*_*H*_ and *W*_*s*_. Some studies suggest that the value of *W*_*s*_ is inversely proportional to the singular value of *S*. Then, it is inferred that the value of *W*_*H*_ is inversely proportional to the singular value of *H*. The specific analysis process is as follows.

Specific steps of Algorithm 2 are as follows.

#### 2.3.2. Evaluation Criteria

In this study, the peak signal-to-noise ratio (PSNR) and structural similarity (SSIM) are used to measure image fidelity, expressed as follows.

(3) $$\begin{array}{c} \text{PSNR}=10\log_{10}\left[\frac{225^{2}}{\left(1/\text{MN}\right)\sum_{i=1}^{M}\sum_{j=1}^{N}{\left(Y_{ij}-X_{ij}\right)}^{2}}\right], \end{array}$$

where *Y*_*ij*_ and *X*_*ij*_ represent the gray values of the reconstructed image and the original image, respectively, and *M*, *N* are the row and column of the image. A higher *PSNR* value indicates better denoising effects.

(4) $$\begin{array}{c} \text{SSIM}\left(x,y\right)={\left[l\left(x,y\right)\right]}^{2}•{\left[c\left(x,y\right)\right]}^{2}•{\left[s\left(x,y\right)\right]}^{2}. \end{array}$$

Based on this, the equation below is obtained.

(5) $$\begin{array}{c} \text{SSIM}\left(x,y\right)=\frac{4\mu_{x}\mu_{y}\sigma_{xy}}{\left(\mu_{x}^{2}+\mu_{y}^{2}\right)\left(\sigma_{x}^{2}+\sigma_{y}^{2}\right)}. \end{array}$$

*l*(*x*, *y*) = (2*μ*_*x*_*μ*_*y*_ + *C*_1_)/(*μ*_*x*_^2^ + *μ*_*y*_^2^ + *C*_1_) represents the comparison of brightness between two images. *c*(*x*, *y*) = (2*σ*_*x*_*σ*_*y*_ + *C*_2_)/(*σ*_*x*_^2^ + *σ*_*y*_^2^ + *C*_2_) represents the contrast comparison of two images. *s*(*x*, *y*) = (*σ*_*xy*_ + *C*_3_)/(*σ*_*x*_*σ*_*y*_ + *C*_3_) represents the structure of the two images. *μ*_*x*_, *μ*_*y*_ and *σ*_*x*_, *σ*_*y*_ represent the mean and variance of *x*, *y*, and *σ*_*xy*_ is the covariance of *x*, *y*.

### 2.4. Statistical Methods

The statistical software SPSS 20.0 was used for data processing. Measurement data are expressed as (*x* ± s), and count data are expressed in cases and percentages. The *χ*^2^ test was carried out for the comparison between different sequences. The consistency test result is described by the Kappa value, and Kappa ≥ 0.7 indicates good consistency, and 0.7 > Kappa ≥ 0.4 indicates acceptable consistency. *P* < 0.05 indicates that the comparison is statistically significant.

## 3. Results

### 3.1. Comparison of Denoising Performance

**Figure 1:**
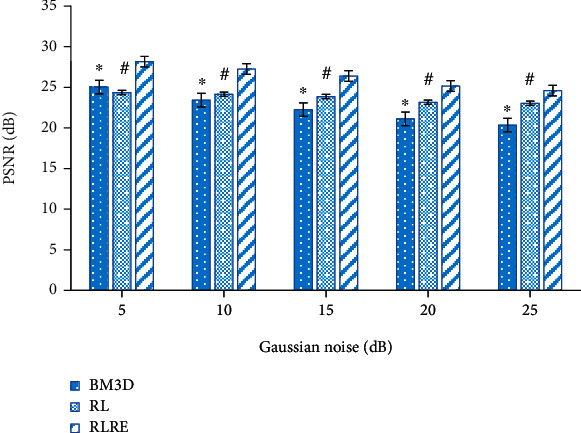
Comparison of PSNR. ∗ indicated that there was a statistically significant difference between the BM3D and the RLRE algorithms (*P* < 0.05); # indicated that there was a significant difference between the RL and RLRE algorithms (*P* < 0.05).

**Figure 2:**
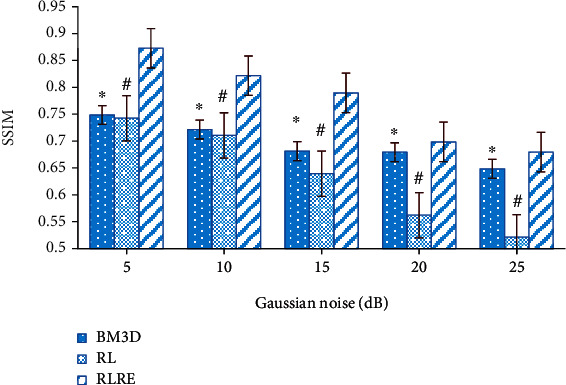
Comparison of SSIM. ∗ indicated the statistical differences between the BM3D and RLRE algorithms (*P* < 0.05), while # meant the same between RL algorithm and RLRE algorithm (*P* < 0.05).

In this study, the denoising effect of the RLRE algorithm was evaluated and compared with the RL algorithm and the block matching 3-D (BM3D) algorithm [22].  Figure 1 shows the PSNR values of images with different levels of noise after processing by the three algorithms. It was noted that as the Gaussian noise increased, the PSNR values of the three algorithms gradually decreased, but the PSHR value of the RLRE algorithm was always higher than the other two (*P* < 0.05).  Figure 2 shows the comparison of SSIM values, and it was noted that the SSIM value of the three algorithms decreased gradually as the Gaussian noise increased, but the SSIM value of the RLRE algorithm was always higher than that of the RL and BM3D algorithms (*P* < 0.05).

**Figure 3:**
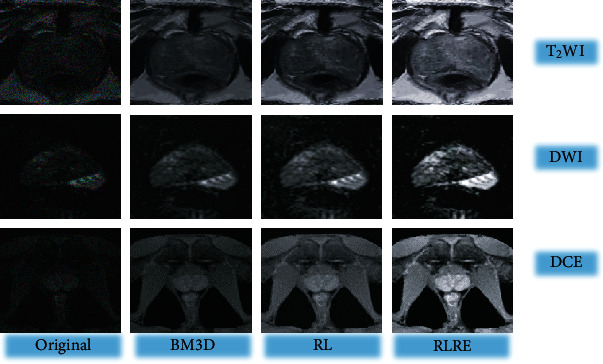
Effects of different algorithms.

Figure 3 shows the image processing effect of BM3D algorithm, RL algorithm, and RLRE algorithm. It was noted that MRI images of T2WI, DWI, and DCE sequences had higher definitions after processing by the RLRE algorithm compared with the original image.

### 3.2. MRI Plain Scan Results

**Figure 4:**
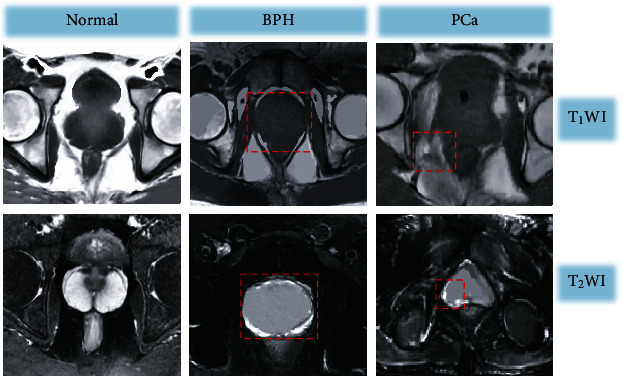
MRI plain scan images. The red circles marked the lesion regions.

Table 1 shows the statistics of the pathological examination results and MRI plain scan examination results. After calculation, it was noted that the sensitivity, specificity, accuracy, and Kappa value of the MRI plain scan sequence for PCa diagnosis were 86.13%, 69.23%, 84.67%, and 0.469, respectively.  Figure 4 shows MRI plain scan images. The examination results of normal prostates were compared with those of BPH patients and PCa patients. Through observation, all of the normal prostate, BPH, and PCa in the T1WI scan sequence showed isosignals, and thus, it was difficult to differentiate from normal prostate tissue. In addition, the central gland was not obviously differentiated from the surrounding belt, so it was impossible to effectively differentiate diseases. T2WI can distinguish the central gland of the prostate from the peripheral zone. In normal subjects, T2WI showed that the peripheral zone was a symmetrical crescent-shaped high signal area, and the signal intensity in the central zone was moderate. For BPH patients, the central gland and transitional zone were obviously enlarged, but the central peripheral zone still maintained a crescent shape with a high signal. For PCa patients, the central area showed a circular surrounding area with mixed high and low signals, and the peripheral zone showed limited low signals.

### 3.3. DWI Examination Results

**Figure 5:**
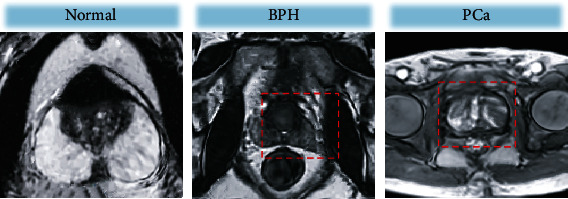
DWI scanning images. The red circles showed the lesions.

Table 2 shows the statistics of pathological examination results and DWI examination results. According to the calculation, the sensitivity, specificity, accuracy, and Kappa value of DWI examination for PCa were 91.97%, 76.92%, 91.33%, and 0.547, respectively.  Figure 5 shows the DWI images. DWI of the normal prostate showed obvious high- and low-signal areas in the central gland and peripheral zone, with clear boundaries. DWI of BPH patients showed a low signal area in the peripheral zone. The transitional zone was obviously enlarged, and the crescent disappeared, while the signal displayed in the central zone was lower, and the central zone could barely be distinguished from the peripheral zone. However, the DWI of PCa showed an uneven high signal area, but the boundary between the central gland and peripheral zone was blurred.

### 3.4. DCE Examination Results

**Figure 6:**
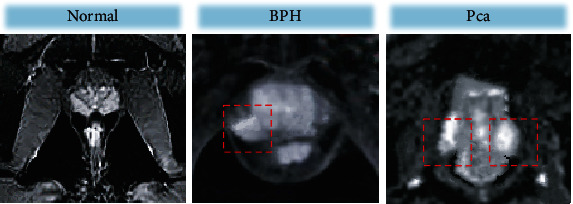
DCE scanning images. The lesion regions were circled by the red circles.

Table 3 shows the statistics of pathological examination results and DCE examination results. According to the calculation, the sensitivity, specificity, accuracy, and Kappa value of DCE examination for PCa were 97.08%, 92.31%, 96.67%, and 0.678, respectively.  Figure 6 showed the DCE images. The structure of normal prostate tissue was clear and showed high signals, while the transitional zone showed low signals, and the central gland was clearly distinguished from the peripheral zone. For BPH patients, DCE showed that the central gland showed high signals, there were hyperplasia lesions with low signals, and a crescent area with a low signal was observed. However, in PCa patients, DCE showed that the central gland was reduced, and the signals in the peripheral zone were uneven, but the boundaries were separable.

### 3.5. Comparison of the Diagnostic Effects of the Three Sequences

**Figure 7:**
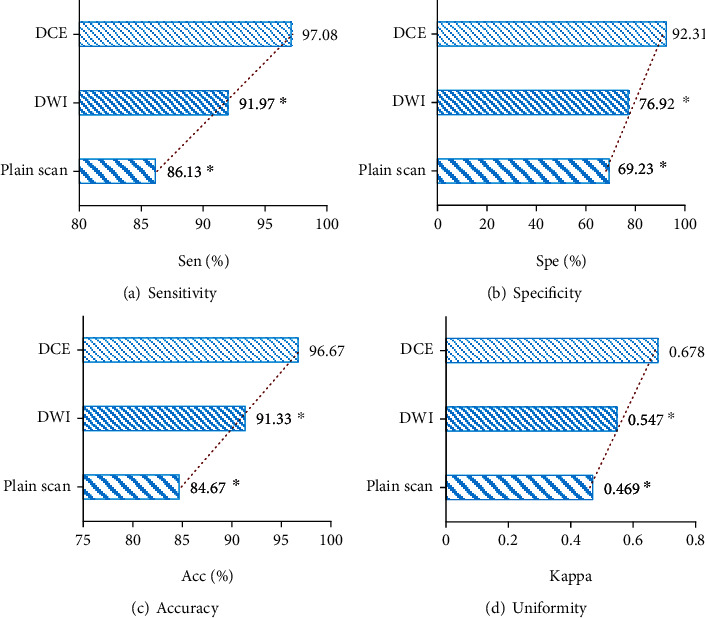
Comparison of the effects of different MRI sequences. ∗ indicated that there was a statistical difference compared with the diagnostic sensitivity, specificity, accuracy, and Kappa value of the DCE sequence (*P* < 0.05).

According to the above results, the sensitivity, specificity, accuracy, and consistency Kappa values of the three MRI scanning sequences were compared, as shown in Figure 7.  Figure 7(a) showed the comparison of sensitivity. The sensitivity of the DCE scan (97.08%) was significantly higher than that of the MRI plain scan (86.13%) and DWI sequence (91.97%) (*P* < 0.05), and DWI was also higher than that of the MRI plain scan (*P* < 0.05).  Figure 7(b) showed the comparison of specificity. The specificity of the DCE scan (92.31%) was significantly higher than that of the plain scan (69.23%) and DWI sequence (76.92%) (*P* < 0.05), and DWI was also higher than that of the MRI plain scan (*P* < 0.05).  Figure 7(c) showed the comparison of accuracy. The accuracy of the DCE scan (96.67%) was significantly higher than that of the plain scan (84.67%) and DWI sequence (91.33%) (*P* < 0.05), and DWI was also higher than that of the plain scan (*P* < 0.05).  Figure 7(d) shows the consistency comparison. The consistency Kappa (0.678) of DCE scan was significantly higher than that of the plain scan (0.469) and DWI sequence (0.547) (*P* < 0.05), and DWI was also higher than that of the plain scan (*P* < 0.05). It was noted that the sensitivity, specificity, accuracy, and consistency of the diagnosis results increased according to the sequence of the MRI plain scan sequence, DWI sequence, and DCE scan sequence, showing statistically significant differences (*P* < 0.05).

## 4. Discussion

With the increase in population, the incidence of PCa is on the rise in China, bringing great pain to the lives of middle-aged and elderly people. Therefore, early diagnosis and early treatment of PCa are very important. At the time of diagnosis, it is inevitable to make a differential diagnosis with other diseases, one of which is BPH. At present, MRI is the main clinical means for the diagnosis of PCa. The study was aimed at evaluating the application effect of MRI in the diagnosis between PCa and BPH.

Specifically, the MRI results of different sequences were compared. The results showed that the diagnostic accuracy of PCa on MRI plain scan was 84.67%, the sensitivity was 86.13%, and the kappa was 0.469, demonstrating good diagnostic discrimination effects, but the specificity was low, only 69.23%. Bai et al. [23] applied imaging screening to the diagnosis of prostate cancer, and the results showed that MRI had a certain sensitivity and specificity. The study of Lewis et al. [24] was consistent with the results of this study, which also suggested that T2WI was feasible, but it still needed to be improved. Some studies have also suggested that the difference between the lesion signal of the normal central gland tissue signal in the surroundings is small, and PCa tissue cannot be well distinguished from normal tissues in the T2WI images of PCa patients [25]. Therefore, in this study, DWI sequences were used for diagnosis, and the results showed that the diagnostic accuracy of DWI for PCa was 91.33%, the sensitivity was 91.97%, and Kappa was 0.547, demonstrating good diagnostic and differential effects, but the specificity was only 76.92%. The research results of Cui et al. [26] also suggested that the DWI sequence can differentiate PCa from BPH lesions by the apparent diffusion coefficient. X. Wang et al. [27] also applied DWI in the invasive differential diagnosis of BPH and low-grade, intermediate-grade, and high-grade PCa and achieved good results, but the premise was the effect of combining it with diffusion kurtosis imaging technology, which indicated that DWI examination technology still had some shortcomings in the differential diagnosis of PCa and BPH. This study found that the accuracy, sensitivity, and Kappa coefficient of DCE in distinguishing PCa from BPH were 96.67%, 97.08%, and 0.678, respectively, which were higher than those of plain scan sequence and DWI sequence. Compared with the first two sequences, the DCE sequence had higher specificity in diagnosis, which indicated that DCE sequence scanning was better in the differential diagnosis of diseases. A large number of research results show that DCE-MRI can distinguish benign and malignant lesions of the prostate [28–30]. Chatterjee et al. [31] proposed that DCE-MRI can distinguish pathological changes well and make a good differential diagnosis between PCa and BPH.

This study was also aimed at further improving the display effect of MRI images. Thus, the RLRE algorithm was introduced to denoise MRI images, and it was compared with the RL algorithm and BM3D algorithm. It was found that the PSNR and SSIM values of the three algorithms gradually decreased with the rise of Gaussian noise, but the PSNR and SSIM values of the RLRE algorithm were always at the highest level among the three. This result suggested that the denoising effect was improved by the RL algorithm. Low-rank theory is the method basis of MRI image processing, and research shows that it has a good application effect [32]. Some studies have suggested that the image restoration algorithm based on the low-rank theory can achieve better application results by weighting [33]. Chen et al. [34] also weighted the RL algorithm with Gaussian noise, and the results showed that the weighted RL algorithm demonstrated a better image processing effect, which supported the results of this study.

## 5. Conclusion

In this study, the RLRE algorithm was introduced to denoise MRI images to differentiate PCa from BPH, aiming to evaluate the diagnostic effect of MRI images with different sequences. The results showed that the RLRE algorithm can improve the display effect and resolution of MRI images. However, RLRE algorithm-based MRI images of DCE sequence were more valuable in the differential diagnosis of PCa and BPH, conducive to the treatment of diseases. Although different sequences of MRI were studied and analyzed in this study, the treatment effect of patients was not analyzed. If this part is added, the research results will be more supportive, and attention should be given paid to this part in subsequent studies. In conclusion, DCE sequence scanning has good application prospects in the differential diagnosis of PCa.

## Figures and Tables

**Algorithm 1:**
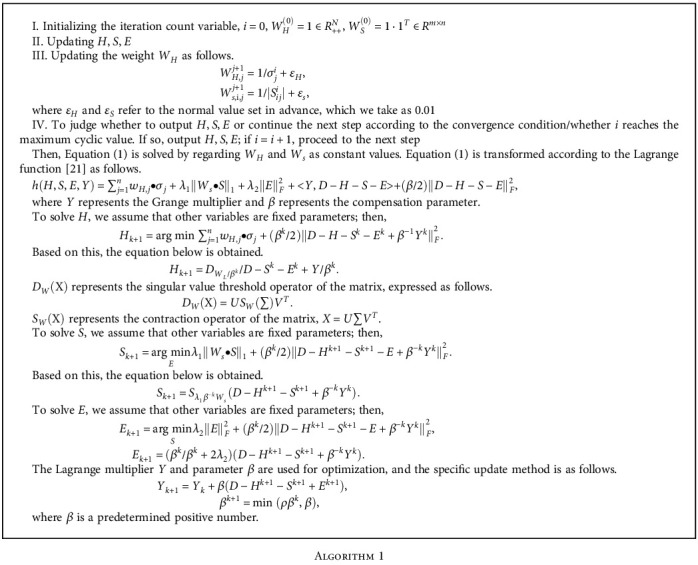


**Algorithm 2:**
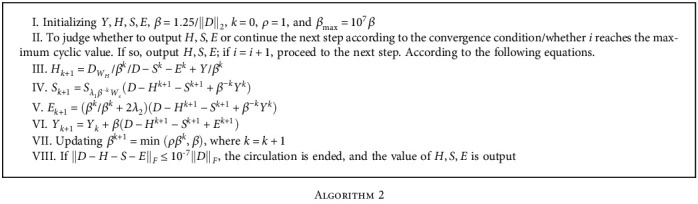


**Table 1:** MRI plain scan and pathological examination result statistics.

		Pathological examination (*n* = 150 cases)		In total
		PCa	BPH	
MRI plain scan (*n* = 150 cases)	PCa	118	4	122
	BPH	19	9	28
In total		137	13	150

**Table 2:** DWI scan and pathological examination result statistics.

		Pathological examination (*n* = 150 cases)		In total
		PCa	BPH	
DWI (*n* = 150 cases)	PCa	126	3	129
	BPH	11	10	21
In total		137	13	150

**Table 3:** DCE scan and pathological examination result statistics.

		Pathological examination (*n* = 150 cases)		In total
		PCa	BPH	
DCE (*n* = 150 cases)	PCa	133	1	134
	BPH	4	12	16
In total		137	13	150

## Data Availability

The data used to support the findings of this study are available from the corresponding author upon request.
